# Minutes of the 17th Annual Meeting of the Miss. Valley Association of Dental Surgeons

**Published:** 1861-03

**Authors:** Geo. Watt


### MINUTES OF THE 17TH ANNUAL MEETING OF THE MISS. VALLEY ASS'N. OF DENTAL SURGEONS.

THE Association met according to adjournment, in the first lecture room of the Ohio College of Dental Surgery, Feb. 20th, 1861, at 10 o'clock, A. M. The President, Dr. Wm. H. Atkinson, was in the chair, and opened the meeting with prayer.

Members present:—Drs. W. H. Atkinson, H. M'Cullum, James Taylor, Charles Bonsall, H. R. Smith, H. A. Smith, T. F. Davenport, J. Richardson, J. W. Baxter, Geo. F. Foote, J. A. McClelland, J. Taft, S. Wardle, and G. Watt.

The Minutes of the last annual meeting were read.

The Executive Committee presented the following report:

The Executive Committee beg leave to offer the following report on the order of business:

I. Reports of Officers.
II. Reports of Committees.
III. Election of Officers.
IV. Essays and Papers.
V. Presentation of new business.
VI. Discussion of the following subjects:
   1. Professional Ethics.
   2. Structure and Nutrition of Dental Tissues.
   3. Reports of Cases—Written or Verbal.
   4. Filling Teeth.
   5. Mechanical Dentistry—Continuous Gum, Vulcanite Base, Gold Work.

G. F. FOOTE,

T. F. DAVENPORT,

STODDARD DRIGGS, } *Committee.*

The report was accepted and adopted.

Other Committees, and the Treasurer, not being ready to report, were excused for the time. On motion of Dr. Watt, the order of business was so modified as to make the election of officers the order of the day for 11½ A. M., to-morrow.

Essays and papers being called for, the President read an essay on "IMPARTATION."

The Treasurer, Dr. C. Bonsall, presented his annual report, and requested that a committee be appointed to audit his account. The report was accepted, and Drs. Taylor and Taft appointed a committee to audit the accounts of the same.

| | | |
|---|---|---|
| Balance in Treasurer's hands at settlement, February 22d, 1860, as audited then | | $204.00 |
| Rec'd since for Initiation Fees and Dues | | 10.00 |
| | | $214.00 |
| 1860. | | |
| Feb. 27, Cash to Janitor, by Society's order | $ 8.00 | |
| 28, " " Reporting Proceedings | 15.00 | |
| Mar. 24, " " Dr. Taft, Dean of College, for Microscope, by Society's order | 100.00 | |
| 1861. | | |
| Feb. 19, Cash, being balance of money lent by order to Publication Committee, in 1855 or '56, and not yet returned | 28.32 | ---151.32 |
| Balance in Treasurer's hands | | $ 62.68 |

Feb. 20th, 1861. C. BONSALL, *Treasurer.*

The auditing committee presented a report as follows:

The committee appointed to audit the Treasurer's accounts, respectfully report that they have examined the same, and find them correct.

J. TAYLOR,<br>J. TAFT, } *Committee.*

Dr. Bonsall also tendered his resignation as Treasurer, and as a member of the Association, in the following communication:

*To the Mississippi Valley Association of Dental Surgeons:*

GENTLEMEN:—As I have been for the last year practically out of the profession, having engaged in other business more suited to my age, and as my interest pecuniarily now requires that I should devote my time and attention to that business, I will ask you to accept my resignation as a member of the Association, and also as your Treasurer; thanking you, at the same time, for the confidence you have shown in me, by electing me to that office each year since the formation of the Society, now seventeen years. I assure you that my interest in this Association, and in every thing which tends to the improvement of Dental Science, will only cease with my life.

Yours respectfully, CHARLES BONSALL.

FEB. 20th, 1861.

It was moved and carried, that this resignation be accepted; and while the motion was pending, Drs. Taylor, Taft, Richardson, Knowlton, Atkinson, Watt and others expressed their regrets at losing a member so earnest, useful and influential as Dr. Bonsall, while, at the same time, they felt it their duty to acquiesce in his course.

On motion of Dr. Taft, Dr. Bonsall was elected an honorary member of the Association.

Adjourned to meet at 2, P. M.

FIRST DAY.—AFTERNOON.

Met according to adjournment, President in the chair.

The remaining committees not being yet ready to report, the reading of essays was again called for. Dr. Atkinson read a paper on "PLATES OVER FANGS."

On motion of Dr. Taft, the subject of this paper was now taken up for discussion.

On motion, all members of the profession, physicians as well as dentists, were invited to participate in the discussions.

The Committee on Membership presented the following names as candidates for membership: Drs. A. E. Lyman, Newton Falls, O.; B. D. Wheeler and Merit Wells, Cincinnati, O.; Geo. S. Allan, Cleveland, O.; who were all unanimously elected.

Adjourned till 9, A. M., to-morrow.

SECOND DAY.—MORNING.

Met according to adjournment, President in the chair.

The Minutes of yesterday were read and approved.

On motion of Dr. Taft, the appointment of delegates to the "American Dental Association" was made the order of the day for 11, A. M., to-day.

The Committee on the Microscope reported as follows:

Your committee would respectfully report that a superior instrument has been purchased, according to the direction of the Association. The cost of the instrument, complete, was $240, $100 of which was paid from your treasury, and $100 from members of the Faculty of the Dental College, and $14 by the Cincinnati Local Society; the balance, $26, has been paid by your committee.

The instrument is in complete order, and has been used for demonstrations to the class in the College, during the past session.

Your committee would suggest the propriety of having mounted, in systematic order, a series of sections of teeth, for the complete exhibition of their anatomical structure. Also, the appointment of a committee, whose duty it shall be to make microscopic investigations upon diseased conditions of the dental tissues, and report upon the same to the next annual meeting.

J. TAFT,

W. H. ATKINSON, } *Committee.*

The report was accepted and adopted, and the Chair was instructed to appoint a committee of three to carry out all its provisions; and, on motion of Dr. Foote, the committee was instructed not to exceed thirty dollars in the expenses of the anatomical specimens thus provided for. Drs. Taft, Richardson, and Atkinson were appointed said committee.

The President, by special request, now read a paper on "DIAGNOSIS."

The regular order of discussions was now taken up, and pending the discussion of No. 1, the time for the appointment of delegates to the "American Dental Association" arrived. It was moved and carried, that the Chair appoint; and, accordingly, the following delegates were appointed: Drs. T. F. Davenport, B. D. Wheeler, Geo. F. Foote, H. M'Cullum, J. A. M'Clelland, and J. Taft. On motion of Dr. Watt, the delegates were empowered to fill vacancies, should any occur.

Proceeded to the election of officers for the ensuing year, which resulted as follows:

H. M'Cullum, *President;* J. Richardson, *Vice-President;* T. F. Davenport, *Corresponding Secretary;* George Watt, *Recording Secretary;* J. Taft, *Treasurer;* H. A. Smith, Geo. Foote, and A. E. Lyman, *Executive Committtee;* J. W. Baxter, W. H. Atkinson, and J. Richardson, *Committee on Membership.*

Adjourned till 2 P. M.

SECOND DAY.—AFTERNOON.

Met according to adjournment. The President appointed Drs. Wheeler and Richardson a committee to conduct the President elect to the chair.

President M'Cullum, on taking the chair, made some interesting and appropriate remarks, thanking the Association for the honor, and giving some practical suggestions tending to expedite business.

Proceeded with the regular order of discussions, and pending the consideration of No. 3, adjourned till 7½ this evening.

SECOND DAY.—EVENING.

Met in accordance with adjournment, President in the chair. Continued the consideration of No. 3, during the entire session. Adjourned till 9, A. M., to-morrow.

THIRD DAY.—MORNING.

Met according to adjournment. On motion, the regular discussions were suspended, to take up some miscellaneous business. On motion of Dr. H. A. Smith, the Treasurer was instructed to pay the sum of twenty dollars, toward paying for the microscope, on condition that the fourteen dollars paid by the Cincinnati Dental Society be refunded. This course seemed necessary, as the original resolution seemed to make no provision for the joint use of the instrument by other societies. The Treasurer was also instructed to pay six dollars to the Janitor for his services to the Association, and ten dollars to the Recording Secretary for reporting the discussions of this meeting.

On motion, the President was instructed to appoint four Essayists for the next annual meeting, and, accordingly, appointed Drs. Geo. F. Foote, H. A. Smith, W. H. Atkinson, and J. Taft.

The Committee on the Prize Essay presented the following report:

Your committee on the Prize Essay would respectfully report that nothing has been done in the way of issuing a new edition of the Essay since the last meeting. The author has not had time to revise the work. There has been some demand for the work, but no supply. It is proposed to have it revised, and a new edition issued as soon as practicable.

J. Taft,
J. Richardson, } *Committee.*

The report was accepted, and the committee re-appointed, with their former instructions.

Resumed the regular discussions, taking up No. 4; and at the close of its discussion, on motion, adjourned to meet at the same place, on Wednesday, February 19th, 1862, at 10 o'clock, A. M. Geo. Watt, *Rec. Sec'y.*

---

APPENDIX, FOR CONVENIENT REFERENCE.

*President*—Dr. H. M'Cullum.

*Vice-President*—J. Richardson.

*Corresponding Secretary*—T. F. Davenport.

*Recording Secretary*—Geo. Watt.

*Treasurer*—J. Taft.

*Executive Committee*—H. A. Smith, G. F. Foote, A. E. Lyman.

*Committee on Membership*—J. W. Baxter, W. H. Atkinson, J. Richardson.

*Delegates to Am. Dental Association*—T. F. Davenport, B. D. Wheeler, G. F. Foote, H. M'Cullum, J. A. M'Clealand, J. Taft.

*Essayists*—G. F. Foote, H. A. Smith, W. H. Atkinson, J. Taft.

*Committee on Microscope*—J. Taft, J. Richardson, W. H. Atkinson.

*Committee on Prize Essay*—J. Taft, J. Richardson.

